# Artificial intelligence skills and their impact on the employability of university graduates

**DOI:** 10.3389/frai.2025.1629320

**Published:** 2025-07-16

**Authors:** Heily Consepción Portocarrero Ramos, Omer Cruz Caro, Einstein Sánchez Bardales, Lenin Quiñones Huatangari, Jonathan Alberto Campos Trigoso, Jorge Luis Maicelo Guevara, River Chávez Santos

**Affiliations:** ^1^Universidad Nacional Toribio Rodríguez de Mendoza de Amazonas, Chachapoyas, Peru; ^2^Pontificia Universidad Católica del Perú, Lima, Peru

**Keywords:** artificial intelligence, employability, university graduates, digital skills, higher education

## Abstract

Artificial intelligence (AI) has emerged as a transformative technology in multiple areas, including the labor market. Its incorporation into organizations redefines professional profiles, required skills, and employability conditions. In this context, it is essential to understand how university graduates are preparing to face these changes and what role their AI skills play in their integration into the workforce. The study aimed to analyze the level of AI skills and their impact on the employability of university graduates through a quantitative and descriptive design. A survey was conducted with a sample of 148 undergraduate and graduate graduates. The data were analyzed using descriptive statistics and visualized using graphs. The results indicated that graduates who report greater knowledge and more frequent use of AI tools, especially generative ones such as ChatGPT, are more likely to be employed in areas related to their majors and to perceive higher productivity and better professional alignment. However, a generational gap in digital skills was also identified, as well as a widespread feeling of insufficient preparation for the challenges of the current labor market. The conclusion is that AI skills are consolidating as a key differentiating factor in employability and that their formal incorporation into university curricula is urgently needed. The implications of the study point to the need for an educational transformation that integrates AI as a transversal skill, promotes ongoing teacher training, and fosters policies that guarantee inclusive education aligned with the challenges of the digital age.

## 1 Introduction

There have been notable advances in artificial intelligence (AI), a technology touted as having significant and perhaps impactful effects on work and jobs (Phan et al., 2017). The emergence of conversational AI systems such as OpenAI’s ChatGPT, Google’s Bard, and Microsoft’s Bing has made this revolution increasingly noticeable, giving rise to a complex discourse about the impact of AI on our work and professional lives (Kessler, 2023). Early reports on how AI will affect the world of work praise AI’s extraordinary potential to boost the global economy (Chui et al., 2023) while warning that AI could affect approximately 80% of workers and potentially displace a quarter of the workforce (Eloundou et al., 2023). Therefore, the impact of AI on people’s careers is significant (Bankins et al., 2024a; Bankins et al., 2024b; Donald et al., 2024). While AI is likely to create new roles and even new industries, it will also fundamentally change or even replace existing jobs, requiring people to develop new skills and making at least some of their existing competencies redundant (Behrend et al., 2024; Selenko et al., 2022). The constant need to continually update skills and knowledge means that individuals’ proactive efforts to develop their skills and shape their careers will likely play an increasingly important role (Hirschi, 2018; Lent et al., 2022).

The adoption of generative artificial intelligence (GenAI) in the workplace is skyrocketing, rising from 22% in 2023 to 75% in 2024 (Feinsod, 2024). This rapid integration has introduced exciting, complex, and profound changes for both employees and organizations (Dwivedi et al., 2023; Dwivedi et al., 2021; Kshetri et al., 2024; Sigala et al., 2024). As expected, the literature on the exciting benefits of using GenAI at work is growing exponentially, documenting its advancement in employee creativity and productivity, as well as improved job satisfaction across a variety of sectors (Bankins et al., 2024a; Bankins et al., 2024b; Dwivedi et al., 2021; Przegalinska et al., 2025; Shao et al., 2024; Voigt and Strauss, 2024). However, the dynamics of employee interactions with GenAI (e.g., collaboration) may be more complex than current optimism suggests (Zirar et al., 2023).

While generative AI has brought with it many new possibilities of AI technology, visible in the workplace (Ramaul et al., 2024), most workers still view AI tools with suspicion and hesitation. For example, a recent survey conducted in Argentina, Denmark, France, Japan, the United Kingdom, and the United States in 2024 found significant differences among internet users regarding awareness and use of generative AI based on age: 56% of 18–24 year-olds say they have used generative AI tools like ChatGPT at least once, compared to 16% of those over 55 (Fletcher and Nielsen, 2024). Furthermore, knowledge of other forms of AI and their applications was found to be very limited, and many employees who are required or permitted to interact with AI tools in the workplace may be inherently resistant to such tools (Golgeci et al., 2025). Related to this, the increasing use of AI tools for greater control and monitoring is another important factor contributing to worker distrust (Monod et al., 2024). Therefore, reluctance and resistance to AI are real problems in the workplace, despite the visibility and use of generative AI tools in recent years (Golgeci et al., 2025).

The introduction of AI technologies into organizations has generated intense debate about their impact on workers and the workplace, with widely polarized opinions. Some suggest it will lead to significant job losses (Frey and Osborne, 2017), while others argue that it will optimize productivity and improve work quality (Jarrahi, 2018; Spencer, 2018). This polarization is exacerbated by broader societal narratives that offer science fiction-based representations of emerging technologies that may mischaracterize current AI systems (Cave et al., 2018). The convergence of these factors can then lead workers to fear the use of AI in their workplaces, regardless of its purpose, and generate negative outcomes for workers, such as lower work engagement, cynicism, and turnover (Brougham and Haar, 2018, 2020). Studies estimate that AI-generated models will affect at least some job tasks for approximately 80% of workers and that a smaller subset of more knowledgeable workers will see most of their functions affected (Eloundou et al., 2023).

With the widespread implementation of AI, human-AI collaboration has become an important and influential employment model. However, there is no consensus in the literature regarding its effectiveness, and little is known about how it affects employee performance (Liu and Li, 2025). In response, the study seeks to analyze AI competencies and their impact on the employability of university graduates. Exploring the impact of AI use on employee behavior is essential because AI has become a technology that drives social progress and impacts various sectors, demonstrating social attributes. Employees play a fundamental role in its application (Liu et al., 2024).

### 1.1 AI and its impact on the workplace

Remarkable advances in AI technologies are not only redefining various aspects of organizational operations but also reshaping work routines, processes, and employee interactions (Brown et al., 2024; Chowdhury et al., 2024). The adoption of GenAI by organizations has intensified this transformation, bringing significant economic and organizational benefits (Bankins et al., 2024a; Bankins et al., 2024b; Dwivedi et al., 2023; Flavián et al., 2022; Voigt and Strauss, 2024). Research on human-AI collaboration suggests that GenAI can provide organizations with sustainable competitive advantages by boosting productivity, improving customer service, enabling the creation of new products, and reducing costs (Kemp, 2024; Raisch and Krakowski, 2021; Wang et al., 2024). This literature predominantly highlights the synergistic benefits of collaboration between employees and GenAI, particularly in fostering positive outcomes of augmented collaborative intelligence (Raisch and Krakowski, 2021). For example, GenAI-assisted employees have been shown to develop more creative solutions to customer queries, driving improvements in sales performance (Jia et al., 2024). Furthermore, this collaboration has been linked to improved employee well-being and productivity, highlighting its potential to positively transform work dynamics (Kong et al., 2023).

The advent of automation and AI in general, and generative AI in particular, is beginning to reshape conventional work models (Kaplan and Haenlein, 2019; Kellogg et al., 2020; Obschonka and Audretsch, 2020; Pachidi et al., 2021). AI helps organizations and individuals achieve new benefits as it increases (Seeber et al., 2020) human tasks, sometimes even replacing them completely (Davenport et al., 2020; Gligor et al., 2021). Recent advances in generative AI, including widely accessible conversational chatbots like ChatGPT, have further accelerated this development while bringing new challenges, doubts, and confusion (Agrawal et al., 2022; Ritala et al., 2024). Both AI-driven automation and scale-up carry the potential for tensions and contradictions among employees (Raisch and Krakowski, 2021), which can contribute to employee hesitancy and resistance to interacting with AI (Golgeci et al., 2025).

Studies have examined the negative outcomes of organizational AI adoption, such as increased job insecurity and decreased willingness to interact with AI (Huang and Gursoy, 2024; Liang et al., 2022; Voigt and Strauss, 2024; Wu et al., 2024; Yin et al., 2024). These findings underscore the potential risks associated with GenAI adoption for both organizations and individuals, including privacy and security concerns, misuse, algorithmic bias, and the exacerbation of the digital divide (Belanche et al., 2024; Gupta and Rathore, 2024; Wirtz et al., 2023). Despite this knowledge, little is known about how and when employee-GenAI collaboration (Kong et al., 2023) can lead to unethical workplace behavior, such as employee convenience (Hai et al., 2025). Expediency, defined as the use of unethical practices to expedite work for selfish purposes (Greenbaum et al., 2018), is a common form of unethical behavior that undermines organizational effectiveness (Hai et al., 2025). For organizational contexts integrated with AI (Eissa, 2020; Xu et al., 2024).

Human-AI collaboration can redefine the future of work, and we assess the associated benefits, challenges, and implications for organizations and their workforces (Przegalinska et al., 2025). The goal is to discern how AI integration can revolutionize organizational perceptions of effective work and the subsequent impacts on productivity, and to highlight how the combined strengths of humans and AI could facilitate effective collaboration (Chalmers et al., 2021; Li et al., 2023; Przegalinska et al., 2019; Sowa et al., 2021; Sowa and Przegalinska, 2020; Townsend and Hunt, 2019), focusing on the synergy between human and artificial intelligence (Hong et al., 2023; Shneiderman, 2022). While generative AI has significant inherent benefits, such as improved work efficiency and creativity, it also poses threats, such as exacerbating concerns about job losses and AI replacement, as well as increasing misinformation, dishonest workplace behavior, and unequal competition (Wach et al., 2023).

Based on human-AI collaboration research (Anthony et al., 2023; Raisch and Krakowski, 2021; Tang et al., 2023), we propose that as GenAI systems increasingly handle daily tasks or automate functions previously performed by humans, employees may feel less engaged in problem-solving or decision-making, diminishing their sense of responsibility for work outcomes. This can foster a separation between self and work, prompting employees to reduce effort or resort to shortcuts (Hai et al., 2025). Human-AI collaboration also changes traditional job characteristics, creating additional strain as employees must continually update their machine skills and handle complex or unverified information when working with GenAI systems (Jia et al., 2024; Shao et al., 2024; Ye and Chen, 2024). These demands can increase work disengagement and encourage disengaging behaviors.

However, previous research suggests that individual perceptions of AI vary widely (Cave and Dihal, 2019; Reina et al., 2025), particularly regarding their integration into the workplace (Bankins et al., 2024a; Bankins et al., 2024b; Selenko et al., 2022). Therefore, the way people react to the impact of AI depends not only on the type of technology implemented but also on individual differences between workers (Bankins et al., 2024a; Bankins et al., 2024b; Lyndgaard et al., 2024). While some initial studies have begun to explore how individual-level factors influence responses to AI, research has primarily focused on how personality influences attitudes toward AI (Kaya et al., 2024; Stein et al., 2024) and how people feel their current job or employment prospects are affected by AI (Bhargava et al., 2021; Lin et al., 2024). However, the way people respond to interactions with AI is profoundly affected by their perceptions of what these experiences might mean for their future (Gioia et al., 1994). However, it is largely unknown how people are more future-focused, career-related cognitions influence their interactions with AI and, therefore, their professional behaviors (Voigt and Strauss, 2024).

The use of AI can significantly change employees’ original tasks and even roles in the workplace, which can increase anxiety and affect engagement (Budhwar et al., 2023; Zerfass et al., 2020). However, when adopted effectively and ethically, generative AI can improve workplace outcomes (Fui-Hoon et al., 2023; Pavlik, 2023). In this sense, there is a pressing need for organizations and leaders to develop strategies to manage the adoption of generative AI, making it positively impact employees and their work experience (Atluri et al., 2024). Despite this, research needs further expansion, especially because AI profoundly affects employee attitudes and behavior (Liu et al., 2024). Drawing on studies on how AI has evolved in the workplace, this study expands the debate on the implications of AI use and how it influences the employability of university graduates.

## 2 Methodology

The research was descriptive and quantitative, to identify and analyze the level of competencies in artificial intelligence (AI) and its relationship with the employability of university graduates.

### 2.1 Data collection

The data collection technique used was a survey, designed with Likert-scale items and multiple-choice questions, initially composed of 20 items. The instrument was validated by expert judgment from professionals with extensive academic and research experience in higher education and digital technologies. Based on their feedback, the questionnaire was refined and reduced to 15 items to enhance clarity, internal coherence, and alignment with the study’s objectives.

To assess the internal consistency of the instrument, Cronbach’s alpha was calculated, yielding a coefficient of 0.876, which indicates a high level of reliability. This value exceeds the commonly accepted threshold of 0.70 for social science research (Ten Berge, 1995; Thorndike, 1995), suggesting that the items are strongly correlated and consistently measure the intended construct. Additionally, the expert validation process ensured the content validity of the instrument, supporting its relevance for measuring university graduates’ perceptions and experiences regarding artificial intelligence (AI) skills and employability (Haynes et al., 1995).

Despite these strengths, certain methodological limitations must be acknowledged. The study relied on self-reported data collected through a cross-sectional design, which may introduce biases such as social desirability, recall inaccuracies, or overestimation of self-perceived competencies (Paulhus and Vazire, 2007; Podsakoff et al., 2003). Moreover, the design does not allow for the establishment of causal relationships between AI competencies and employability outcomes (Shadish et al., 2003). To reduce potential bias, participant anonymity and written informed consent were ensured. However, future studies should consider using longitudinal designs, triangulated data sources, or objective performance-based assessments to enhance the internal and external validity of findings (Creswell and Plano, 2017).

The study population consisted of undergraduate and graduate alumni from the National University Toribio Rodríguez de Mendoza of Amazonas (UNTRM-A). A total of 174 respondents completed the survey, but after excluding blank or irrelevant responses, a final sample of 148 participants was obtained through non-probabilistic convenience sampling. The instrument was administered in April 2025, and all participants provided written informed consent, authorizing the academic use of the collected data.

All participants gave their written informed consent to participate in the study, ensuring the publication of the data.

### 2.2 Data analysis

The data were analyzed using descriptive statistics. These data were run and analyzed using RStudio software. Graphical analysis was also used to facilitate the interpretation of the relationships between AI competencies and employability variables.

Table 1 describes the participants’ data and employment status. The sample is composed of 148 university graduates, with a majority of women (57.4%) versus men (42.6%). Regarding age, the majority is in the 26–30 age range (36.5%), followed by the 21–25 age group (24.3%) and the 31–35 age group (22.3%). This indicates that the sample is composed primarily of young and early middle-aged professionals, with a much smaller presence of individuals over 40 years of age.

**Table 1:** Socioeconomic data.

Variables	Frequency	Percentage
Gender		
Female	85	57.4
Male	63	42.6
Age		
21 to 25	36	24.3
26 to 30	54	36.5
31 to 35	33	22.3
36 to 40	15	10.1
41 to 45	6	4.1
46 to 50	3	2.0
50 or more	1	0.7
Year of graduation		
2007 to 2010	12	8.1
2011 to 2015	20	13.5
2016 to 2020	39	26.4
2021 to 2025	77	52.0

Regarding the year of graduation, more than half of the participants (52%) are recent graduates (2021–2025), followed by those who graduated between 2016 and 2020 (26.4%). This suggests that most respondents have relatively recent professional experience, which is relevant considering that the rise of AI in the workplace has intensified in recent years.

Table 2 shows the graduates’ employment situation. Regarding their current employment status, the majority (58.8%) work in sectors related to their degree, indicating a good match between training and employment. However, a significant group (22.3%) is currently seeking employment, reflecting a certain level of unemployment among graduates. It is also notable that 14.9% work in sectors unrelated to their degree, suggesting that some graduates have had to diversify their careers.

**Table 2:** Employment-related variables.

Variables	Frequency	Percentage
Employment status		
Looking for a job	33	22.3
Entrepreneurship	6	4.1
Working in the sector related to my career	87	58.8
Working in a sector unrelated to my career	22	14.9
Work experience		
I have never worked until now.	7	4.7
I have only worked for short periods.	20	13.5
I have alternated periods of employment and unemployment.	40	27.0
I have been employed most of the time.	80	54.1
Own business	1	0.7
The most determining factor in getting a job		
I have never worked until now.	3	2.0
University education	33	22.3
Previous experience or internship	59	39.9
Specializations	15	10.1
Network of contacts or recommendations	38	25.7

Regarding work experience, the majority (54.1%) have held stable jobs, having been employed most of the time. Twenty-seven percent have alternated periods of employment and unemployment, indicating a degree of job instability for more than a quarter of those surveyed.

Regarding the most important factor in finding a job, previous experience or internships stands out as the main factor (39.9%), followed by a network of contacts or recommendations (25.7%) and university education (22.3%). This suggests that practical knowledge and social capital carry more weight than academic qualifications when it comes to finding a job.

## 3 Results

**Figure 1:**
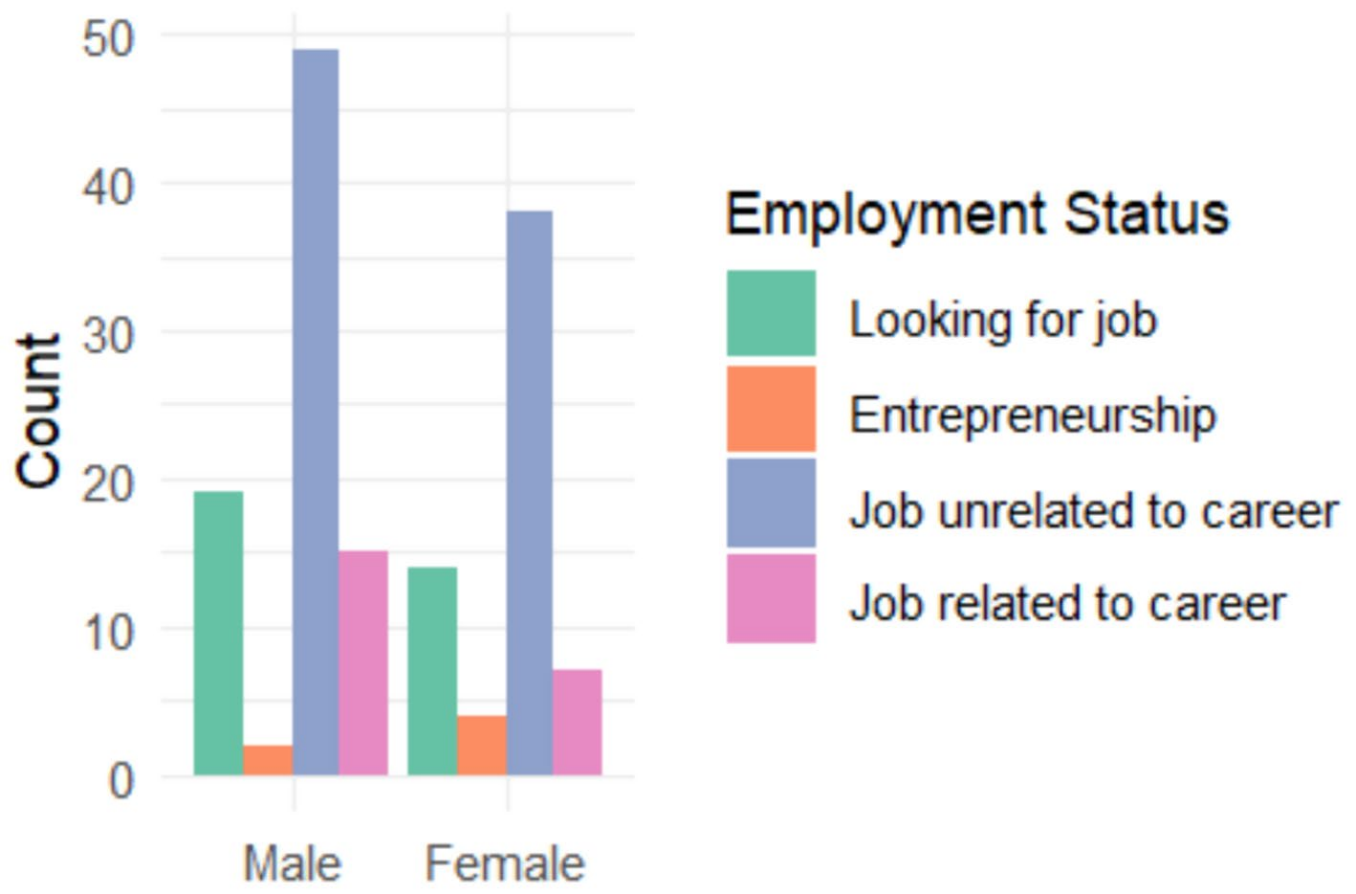
Employment situation by gender of university graduates.

Figure 1 shows a clear distribution of employment status by gender. It is observed that both men and women present similar patterns, with the majority working in sectors related to their degree. However, there is a slight proportional difference: women appear to have a slightly higher employment rate in sectors related to their academic training. It is also evident that there is a considerable number of graduates of both genders seeking employment, with this percentage being similar for men and women. Entrepreneurship appears to be the least common option for both genders, suggesting that most graduates prefer the security of formal employment over starting their businesses.

**Figure 2:**
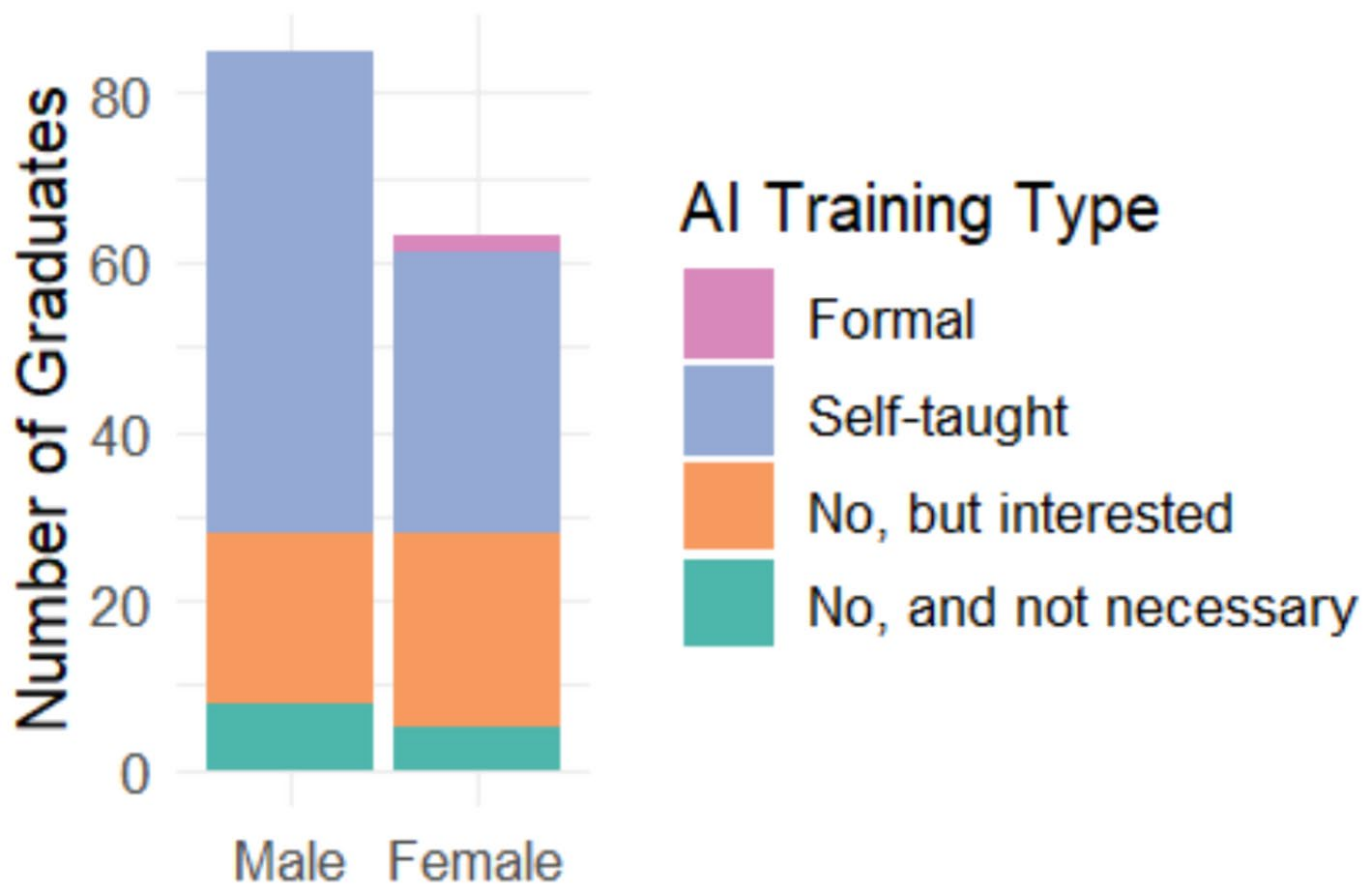
Type of AI training received by graduates.

Figure 2 reveals how graduates have acquired AI knowledge. Self-directed learning stands out as the predominant method, suggesting considerable personal initiative among graduates to stay up-to-date with AI technologies. Online courses represent the second most common route, reflecting the importance of digital learning platforms. It is striking that formal university training in AI appears to be a relatively low percentage, which could indicate a gap between university curricula and current labor market demands regarding AI skills.

**Figure 3:**
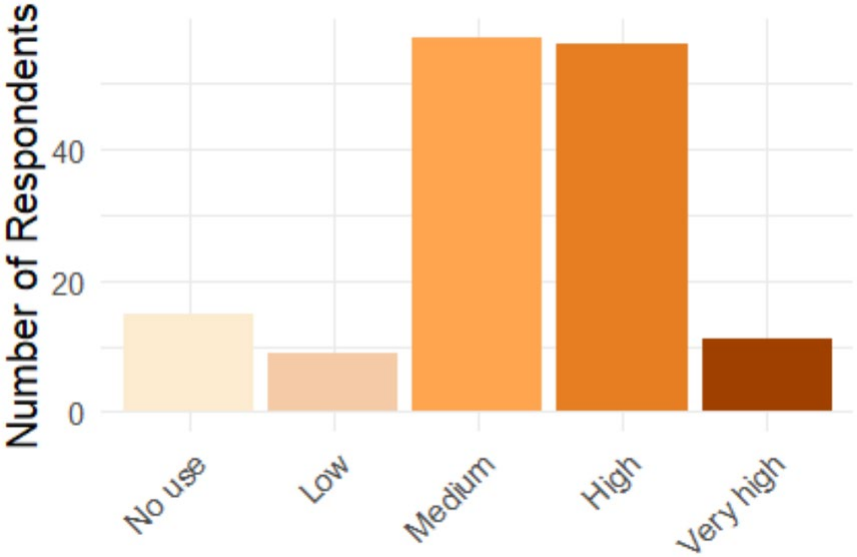
Level of confidence in AI tools among graduates.

Figure 3 shows a varied but positive distribution of trust in AI tools. Most graduates fall into the “moderately trustworthy” and “very trustworthy” categories, indicating a generally positive perception of these technologies. However, a significant segment maintains a neutral or skeptical stance. This pattern suggests that, while there is growing acceptance of AI, some graduates still have doubts or reservations, possibly related to concerns about privacy, ethics, or the impact of AI on their careers.

**Figure 4:**
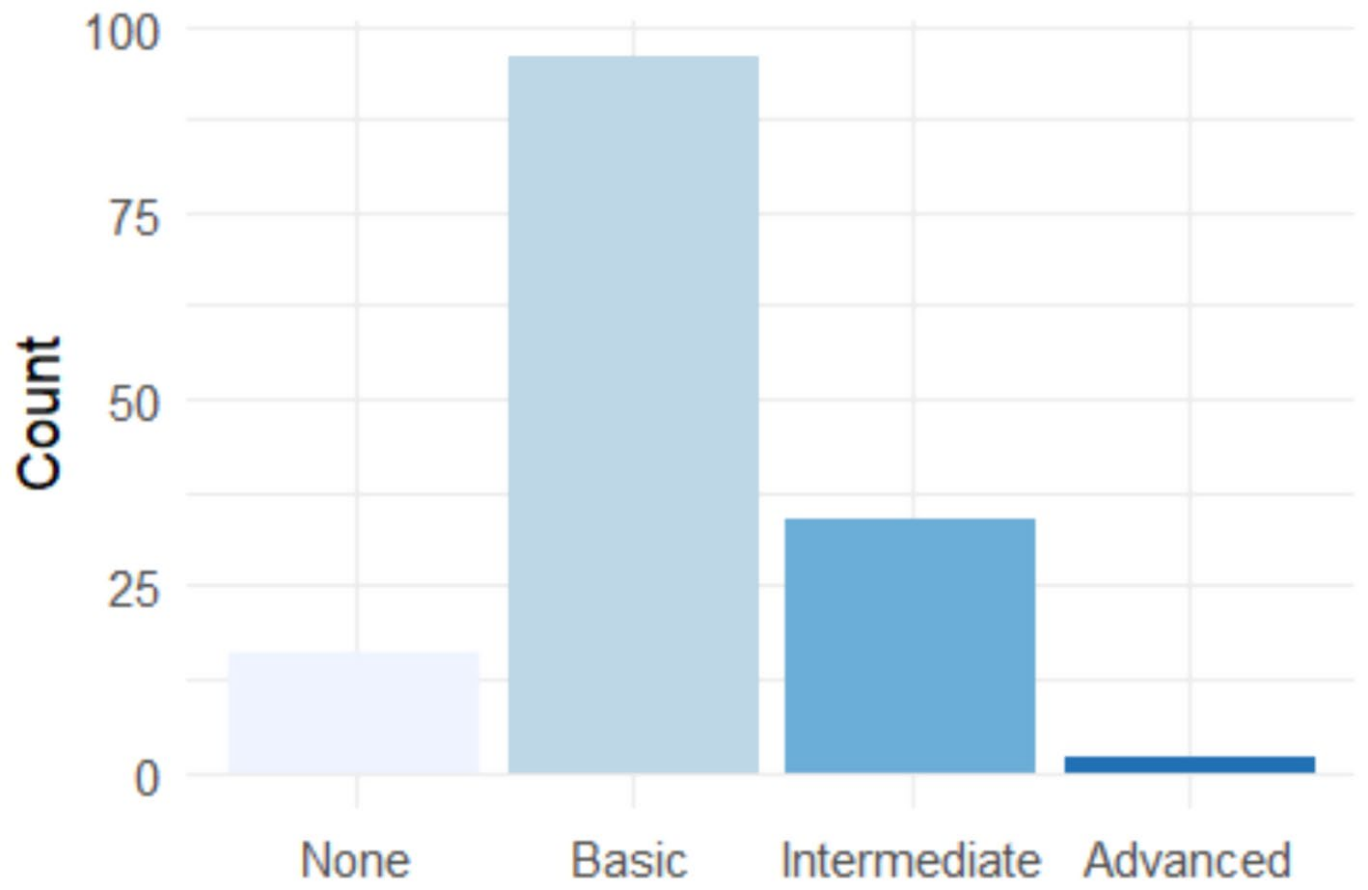
The graduates’ level of knowledge about AI tools for their application in the employment field.

Figure 4 shows a normal distribution of knowledge of AI tools applied to the workplace. The majority of graduates are concentrated at an “intermediate” level of knowledge, with smaller proportions at the “beginner” and “advanced” extremes. This distribution suggests that there is a general knowledge base, but that there is still considerable room for the development of more advanced AI skills. Interestingly, very few graduates consider themselves “experts,” which could represent an opportunity for differentiation in the labor market for those who delve deeper into these skills.

This is also because, for the most part, graduates report using accessible and easy-to-use tools more frequently, such as ChatGPT (OpenAI), followed by Google Gemini and Microsoft Copilot, among the most frequently mentioned. This demonstrates that graduates are primarily familiar with general-purpose generative AI tools but have not yet explored or mastered more technical or specialized applications, such as those related to data analysis, process automation, computer-aided programming, or intelligent solution design.

Furthermore, this pattern suggests that AI knowledge is more closely tied to practical and intuitive experience than to formal technical training. While this facilitates initial adoption, it can also limit professional growth if not accompanied by systematic and in-depth training. From a labor perspective, this implies that graduates with more advanced knowledge, for example, in machine learning, natural language processing, or AI integration into workflows, have a clear advantage over those who only master basic tools. Therefore, deepening their understanding of the strategic and specialized use of AI can be a key way to improve employability and access positions of greater responsibility and better pay.

**Figure 5:**
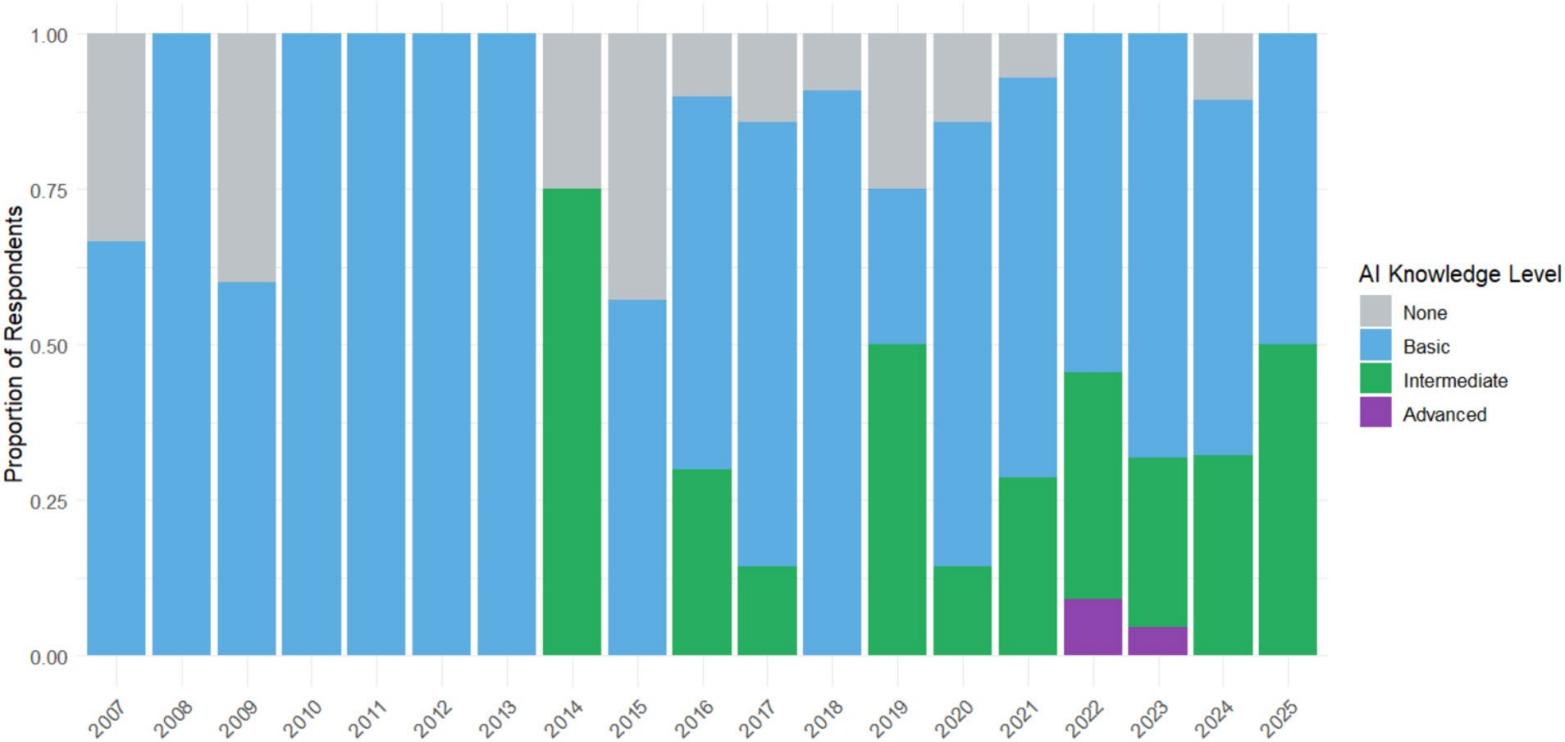
Level of AI knowledge among graduates according to the year of graduation from the university.

Figure 5 establishes a clear temporal relationship: the most recent graduates (2021–2025) show higher levels of AI knowledge compared to previous generations. This reflects how the integration of AI into university curricula has evolved. Graduates from earlier periods (2007–2010) mostly show basic or beginner levels, while intermediate generations show a gradual transition. This pattern suggests the growing importance that AI has acquired in university education in recent years, as well as the need for refresher courses for graduates from previous classes.

This pattern confirms the growing importance of AI in vocational training but also highlights a crucial issue: the generational gap in digital skills. While younger people are entering the labor market with better technological tools, many more experienced professionals may be left behind if they do not continually update themselves.

Regarding their perception of the importance of AI, 53.4% consider it important for their professional field, and 46.0% believe it is very important. Only 0.6% consider it to be of little relevance. This demonstrates a nearly unanimous awareness of the key role AI plays in today’s professional practice, regardless of the area of training. The majority of graduates no longer view AI as a fad but as a necessary tool for competing, innovating, and staying current in the workforce.

Furthermore, when asked whether universities should strengthen AI teaching across all majors, 87.2% responded affirmatively, while 12.8% believe it should be taught only in certain disciplines. This demonstrates a clear and urgent demand from graduates toward educational institutions: AI should not be viewed as a subject exclusive to engineering or technology, but rather as a transversal skill, useful for doctors who analyze health data, lawyers who write with AI assistance, or educators who personalize learning.

These results not only show an evolution in AI knowledge over time but also a strong expectation that universities modernize and prepare their students for a market where AI is already a key player. Continuous updating is not just an advantage; it’s a necessity to ensure students do not miss out on current and future job opportunities.

**Figure 6:**
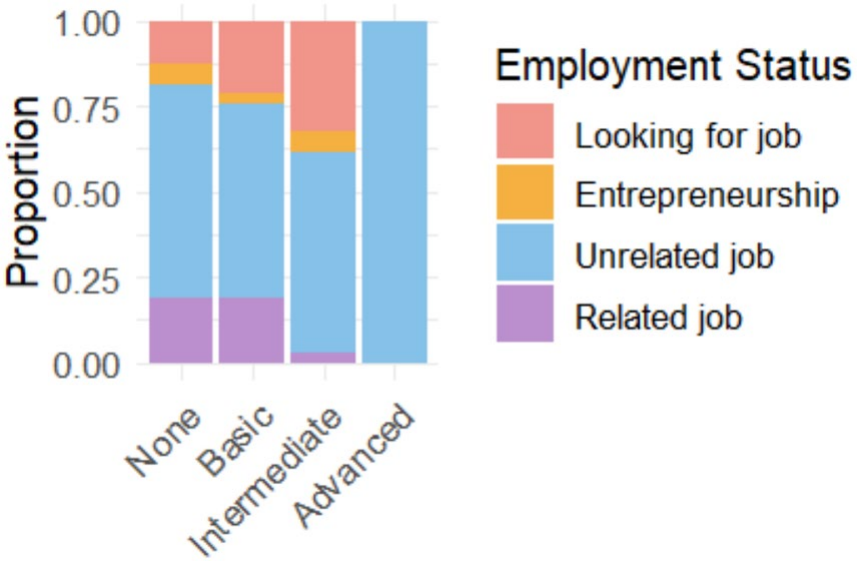
Relationship between the level of knowledge of AI and the current situation regarding the employment of graduates.

Figure 6 shows a significant correlation between AI proficiency and employment status. Graduates with advanced or expert AI skills are more likely to be employed in jobs related to their field of study, while those with basic knowledge have higher unemployment rates or are employed in sectors unrelated to their training. This trend suggests a potential association between AI skills and more favorable employability outcomes, suggesting that investing in developing these skills can translate into better job opportunities and greater alignment between training and employment.

**Figure 7:**
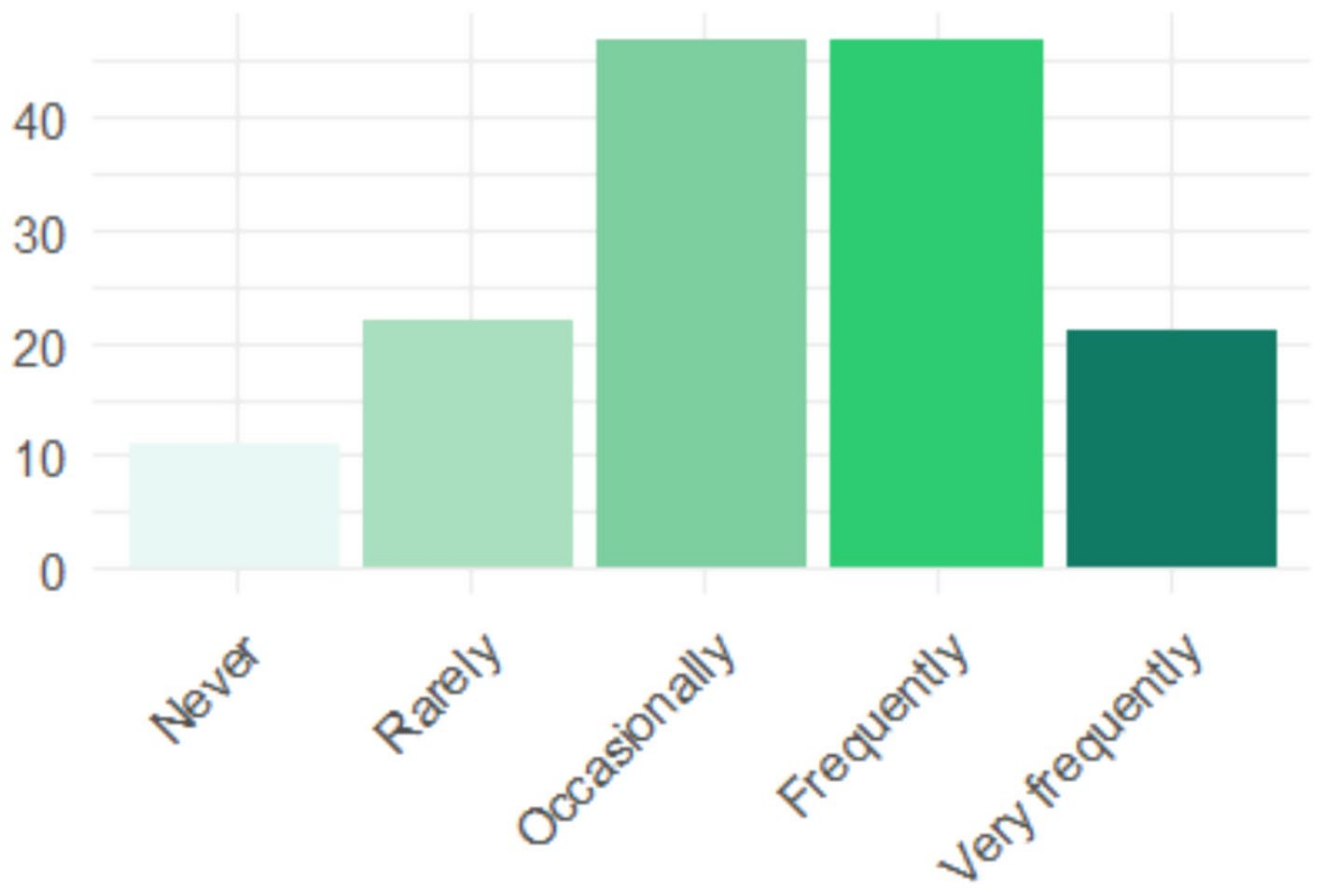
Frequency of AI use in employment by graduates.

Figure 7 reveals patterns of AI tool use in work contexts. Moderate use predominates (several times a week), followed by daily use. It is notable that few graduates report never using these tools, confirming the growing penetration of AI in various professional environments. This distribution suggests that AI is becoming a common work tool, although it has not yet reached the level of daily use for all professionals.

**Figure 8:**
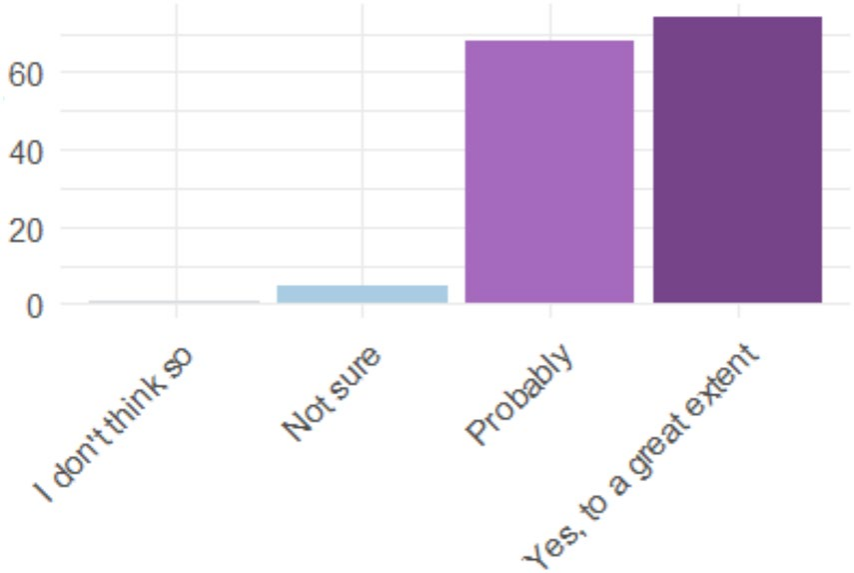
Perception of whether knowledge and application of AI tools will increase job opportunities.

Figure 8 shows a broad consensus among graduates: the vast majority perceive that knowledge and application of AI tools will increase their job opportunities, with a strong concentration of “agree” and “strongly agree” responses. This positive perception indicates that graduates recognize the strategic value of these skills for their professional future. The low number of negative responses reinforces the idea that there is widespread awareness of the importance of adapting to a labor market increasingly influenced by AI.

**Figure 9:**
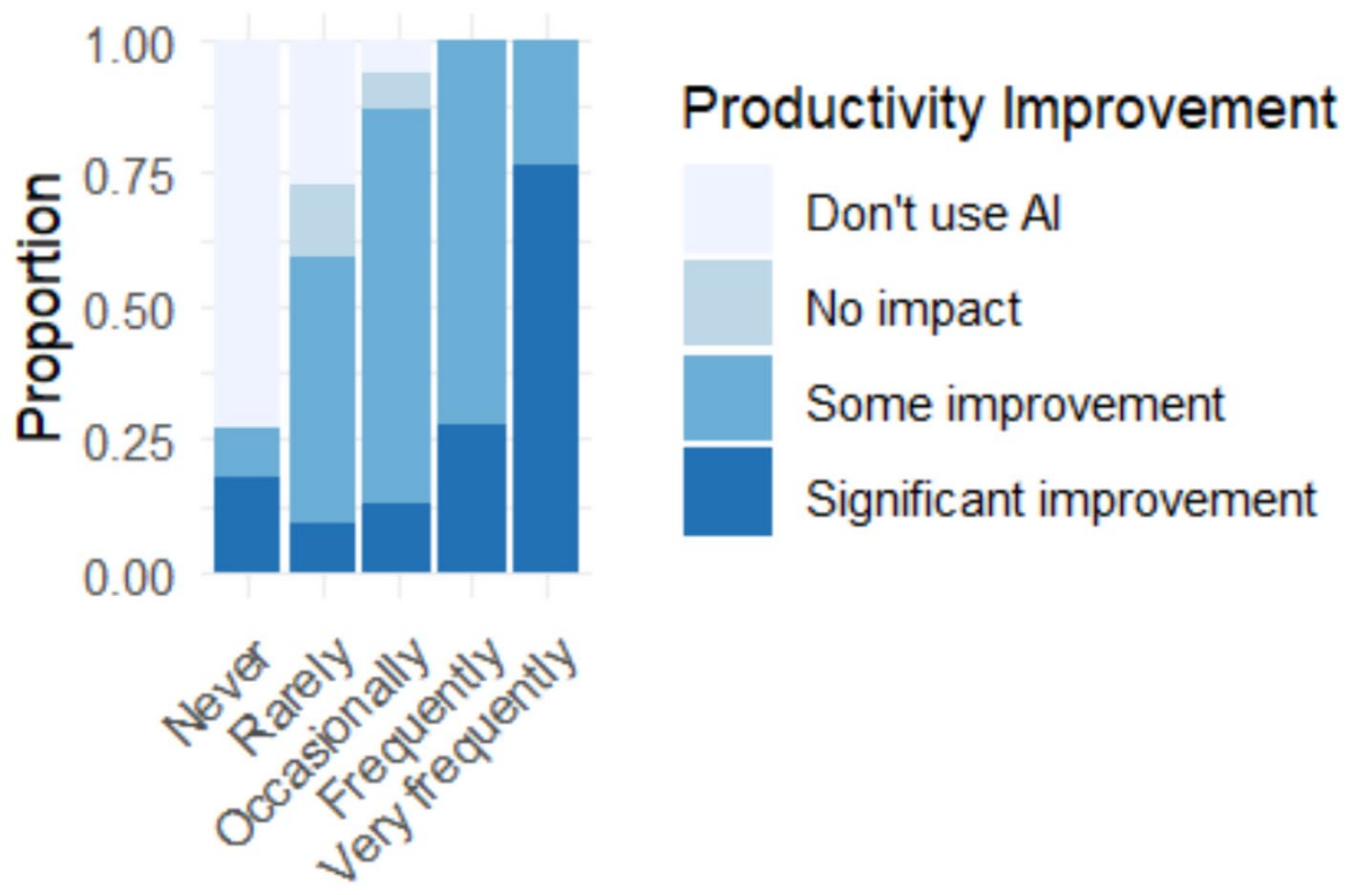
Relationship between the frequency of use of AI tools and how these have improved work productivity.

Figure 9 establishes a direct correlation between the frequency of use of AI tools and the perception of improvements in work productivity. Frequent users (daily or several times a week) report greater improvements in their productivity, while those who use these tools less frequently perceive more limited benefits. This positive relationship may reflect an association between greater exposure to AI tools and perceived improvements in productivity; the greater the exposure and practice with AI tools, the greater the ability to take advantage of their benefits in terms of efficiency and work performance.

Graduates who use these tools daily or several times a week report greater perceptions of efficiency, speed in task execution, and improved work quality, compared to those who use them occasionally or rarely. This suggests a clear progressive learning effect: the more these technologies are used, the more they understand their potential and learn to strategically integrate them into daily work.

Furthermore, when graduates were asked how prepared they feel to face changes in the labor market due to AI, 52.0% said they were “somewhat prepared,” 26.7% felt “little prepared,” only 16.9% said they were “very prepared,” and 4.7% admitted to feeling unprepared at all. This data reflects a widespread feeling of insufficient training, which reinforces the need for AI education not to be optional or extracurricular, but rather a structural part of the educational process from the first cycles of university studies. Preparing students for the critical, ethical, and practical use of these technologies is essential if we want to train professionals who can adapt to a constantly evolving work environment.

On the other hand, 95.6% of graduates expressed interest in continuing to take courses on AI tools to strengthen their professional performance. This figure not only demonstrates motivation and proactivity but also highlights a significant opportunity for universities and continuing education centers: designing specialized programs, diplomas, or workshops that respond to this growing demand.

These results, taken together, present a clear and compelling picture of AI skills emerging as a key differentiating factor in the employability of university graduates. Those who have developed more advanced AI knowledge are more likely to access jobs related to their field of study and feel more productive, confident, and prepared to face changes in the world of work.

However, it is also evident that many graduates still need support, formal training, and real opportunities to acquire these skills. Therefore, the call is clear: universities must take an active role in AI training, and educational policies must promote an education more connected to the demands of the present and the future. Only in this way will we achieve a fair, inclusive, and effective transition to a labor market increasingly driven by artificial intelligence.

**Figure 10:**
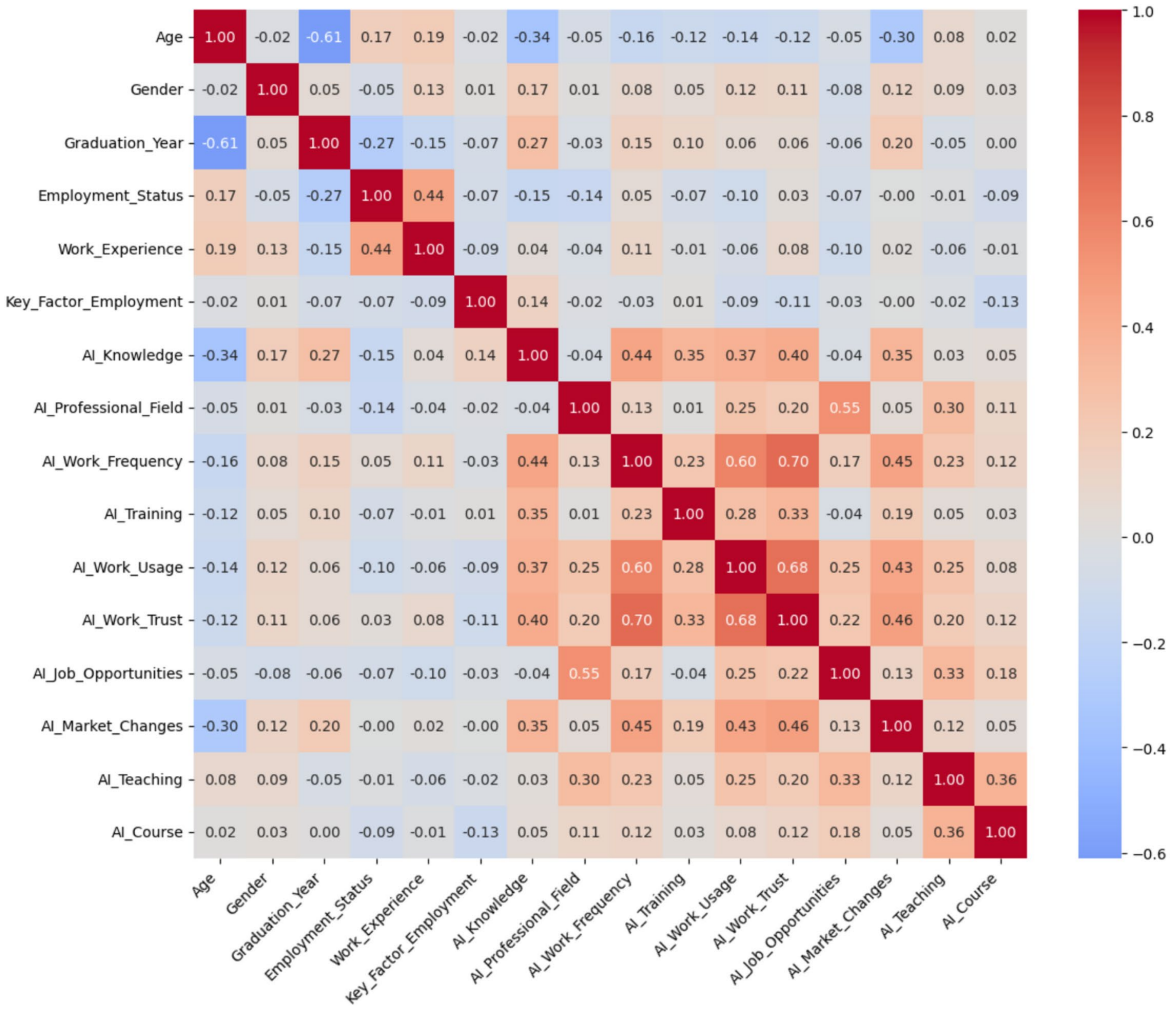
Pearson correlation matrix among sociodemographic variables, AI competencies, and employability perceptions.

The Pearson correlation matrix (Figure 10) shows linear associations between sociodemographic variables, AI-related competencies, and perceptions of labor impact. Moderate positive correlations were found between AI usage at work (AI_Work_Usage) and trust in AI (AI_Work_Trust, r = 0.68), as well as with usage frequency (AI_Work_Frequency, r = 0.60). There is also a notable correlation between the relevance of AI to one’s professional field (AI_Professional_Field) and the belief that AI increases job opportunities (AI_Job_Opportunities, r = 0.55), suggesting that those who find AI applicable in their careers also foresee emerging opportunities.

Additionally, AI knowledge (AI_Knowledge) correlates positively with usage frequency (r = 0.44), trust (r = 0.40), and perceived market changes (r = 0.35). In contrast, age shows negative correlations with AI knowledge (r = −0.34) and market changes (r = −0.30), indicating that younger graduates report greater familiarity and sensitivity toward the implications of AI in the labor market.

These relationships support the structural validity of the instrument and justify the application of Principal Component Analysis (PCA) to uncover latent patterns in perceptions and skills.

**Figure 11:**
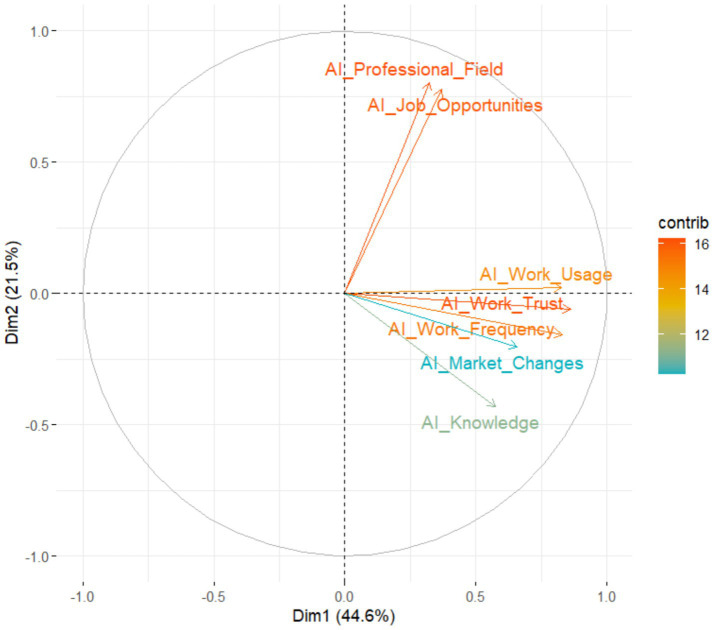
Principal component analysis (PCA).

The Principal Component Analysis (PCA) shown in Figure 11, based on variables related to the knowledge, use, and perception of artificial intelligence (AI) in the professional environment of university graduates, allows us to visualize how these factors interrelate within a reduced two-dimensional space. This type of analysis aims to synthesize information into components that explain most of the variability in the responses, helping us to understand underlying patterns in participants’ perceptions and experiences with AI.

The first principal component (Dim1), which accounts for approximately 45% of the total variability, is strongly influenced by variables such as the perceived importance of AI in the professional field (AI_Professional_Field), the belief that AI will increase job opportunities (AI_Job_Opportunities), and the actual use of AI tools in the workplace (AI_Work_Usage). This suggests that graduates who consider AI a crucial tool in their profession are also those who tend to use it more frequently and who hold positive expectations about its impact on employability. In other words, there is a distinct group of professionals who not only value AI from a strategic perspective but also apply it in their daily work life.

The second component (Dim2), which explains around 21% of the variability, is more closely related to the self-reported level of AI knowledge (AI_Knowledge) and the perceived preparedness to face market changes driven by AI (AI_Market_Changes). The positioning of these variables suggests that technical knowledge, while important, is not necessarily aligned with actual use or perceived opportunities in the labor market. This may point to a gap between academic training in AI and its practical application in real-world work environments. In other words, some graduates may feel technically capable, but not necessarily prepared to confront the challenges posed by digital transformation.

This finding is consistent with the results of the applied survey, which revealed that many graduates have received some type of AI training, either formal or self-taught, and consider this competence important or very important for their professional development. However, an uneven implementation of AI in the workplace is also observed, as well as varying levels of confidence and readiness to face a labor future influenced by such technologies.

Overall, the PCA supports the idea that AI-related employability depends not only on technical knowledge but also on attitudinal factors, personal perception, and the professional context. Universities, therefore, face both the challenge and the opportunity of strengthening AI education within their academic programs. This involves not only the inclusion of technical content but also preparing future professionals to interpret, apply, and lead changes in their fields, driven by digital transformation.

Table 3 presents the results of the Principal Component Analysis (PCA), specifically the percentage of variance explained by each extracted component. The first component explains 44.6% of the total variance, and the second component explains 21.5%, resulting in a cumulative total of 66.1% of the explained variance in variables related to AI competencies and perceptions. This outcome is methodologically robust, as it is generally accepted that the first two components should explain more than 60% of the variance in social and educational research.

**Table 3:** Percentage of variance explained by the principal components from the analysis of AI competencies and perceptions.

Dimension	Explained variance (%)	Cumulative variance (%)
Dim1	44.57	44.57
Dim2	21.54	66.11
Dim3	9.18	75.29
Dim4	8.93	84.22
Dim5	6.42	90.64
Dim6	5.47	96.11
Dim7	3.89	100.00

This finding suggests that a large portion of the complexity of the phenomenon can be summarized into two main dimensions, validating the dimensionality reduction and allowing for the identification of latent patterns in graduates’ responses regarding the use, knowledge, and perceptions of AI in professional settings.

Table 4 shows the rotated factor loadings of each variable on the two principal components extracted through PCA. These loadings represent the degree of association of each variable with the underlying components. A varimax rotation was applied to improve interpretability.

**Table 4:** Rotated factor loadings of principal components extracted through PCA.

Variable	Dim1	Dim2	Dim3	Dim4	Dim5	Dim6	Dim7
AI_Professional_Field	0.183	0.655	−0.177	0.190	−0.630	−0.272	−0.010
AI_Job_Opportunities	0.209	0.633	0.128	0.223	0.681	0.161	0.007
AI_Work_Usage	0.469	0.019	−0.109	−0.297	−0.210	0.723	−0.336
AI_Work_Trust	0.488	−0.050	−0.097	−0.343	0.041	−0.092	0.789
AI_Work_Frequency	0.469	−0.127	−0.147	−0.259	0.234	−0.599	−0.511
AI_Market_Changes	0.372	−0.166	0.848	0.271	−0.194	−0.061	−0.016
AI_Knowledge	0.326	−0.352	−0.436	0.754	0.043	0.080	0.057

The first component clusters variables such as AI_Work_Usage, AI_Work_Trust, AI_Work_Frequency, AI_Training, AI_Knowledge, and AI_Professional_Field. These variables reflect operational competencies, trust, training, and experience with AI in work contexts. Therefore, this component can be interpreted as representing “Functional competencies in artificial intelligence”.

The second component is primarily associated with AI_Job_Opportunities and AI_Market_Changes, which capture perceptions regarding the impact of AI on the labor market. Hence, this component reflects a dimension that can be referred to as “Perceived impact of AI on employability”.

This factorial structure supports the construct validity of the instrument and enables the classification of graduates based on their skill profiles and expectations related to AI.

The T-test (Table 5) examined whether there are significant differences in AI-related skills and perceptions between men and women. The only statistically significant result was found in the “AI knowledge” variable (*p* = 0.0393), with women reporting lower average knowledge levels. This points to a gender gap in AI proficiency, potentially linked to disparities in access, confidence, or prior exposure to digital tools. In the remaining variables (AI usage, trust, relevance to career, job opportunities, and perceived market changes), no significant differences were detected, suggesting that men and women generally share similar perceptions of AI’s impact on their employability, even if they differ in technical knowledge.

**Table 5:** T-test by gender.

Variable	*t*-statistic	*p*-value
AI_Knowledge	−2.082	0.0393
AI_Work_Usage	−1.402	0.1634
AI_Work_Trust	−1.368	0.1736
AI_Professional_Field	−0.153	0.8783
AI_Job_Opportunities	0.996	0.3209
AI_Market_Changes	−1.536	0.1269

The ANOVA test explored whether AI-related skills and perceptions vary according to graduates’ current employment status (employed in-field, out-of-field, unemployed, and entrepreneurs). The analysis found no statistically significant differences among the groups (Table 6). This indicates that graduates’ levels of AI knowledge, usage, and perceptions are relatively uniform across employment categories. Although some trends were observed, such as slightly higher AI usage among those employed in their field, they were not strong enough to be statistically validated. These findings may suggest that the practical role of AI in employability has not yet fully materialized or that external factors like internships or professional networks still weigh more heavily in job placement.

**Table 6:** ANOVA by Employment Status.

Variable	*t*-statistic	*p*-value
AI_Knowledge	1.619	0.1876
AI_Work_Usage	1.125	0.3412
AI_Work_Trust	0.282	0.8381
AI_Professional_Field	1.293	0.2792
AI_Job_Opportunities	0.398	0.7545
AI_Market_Changes	0.148	0.9309

## 4 Discussion

The results of this study reflect the radical transformation that artificial intelligence (AI) is causing in professional profiles and employment dynamics, as anticipated by Phan et al. (2017), who warned that AI would have a significant impact on the structure of work. The fact that graduates with greater mastery of AI tools report greater job placement and better alignment with their professional field supports the idea that these skills may be increasingly important in the labor market. This finding coincides with that indicated by Eloundou et al. (2023), who estimate that around 80% of workers will see at least some of their tasks modified due to the advancement of language models such as GPT. AI, therefore, not only redefines existing jobs but also introduces new ways of working, demanding a constant reconfiguration of skills and roles (Behrend et al., 2024; Selenko et al., 2022).

In this sense, this confirms the arguments put forward by Bankins et al. (2024a), Bankins et al. (2024b), and Donald et al. (2024), who argue that the workers of the future will require not only technical skills but also sustained proactivity in managing their careers. The fact that the majority of graduates surveyed in this study stated that they felt only “somewhat prepared” or even “little prepared” to face the changes derived from AI reveals a worrying gap between the evolution of the labor market and the actual preparation offered by educational environments. This coincides with the concern of Hirschi (2018) and Lent et al. (2022) about the need to promote autonomous and continuous learning as a fundamental axis of professional sustainability.

Furthermore, the predominance of self-learning and online courses as the main training routes in AI reinforces the findings of Dwivedi et al. (2021) and Fletcher and Nielsen (2024), who identify that younger professionals are taking the lead in the adoption of AI tools, particularly through digital platforms and not necessarily through structured training at university. This situation, however, reflects a significant curricular gap, as Reina et al. (2025) and Ritala et al. (2024) warn, many universities have not yet incorporated AI transversally into their curricula, leaving students and graduates adrift in the face of changes in the environment.

Another key finding of this study is the strong relationship between the frequency of AI tool use and the perception of improved productivity. Graduates who regularly use these technologies report greater efficiency, creativity, and responsiveness, which is fully in line with the findings of Jia et al. (2024), who document that human-AI collaboration significantly improves employee performance, especially in complex tasks. Similarly, Kong et al. (2023) and Shao et al. (2024) show how sustained use of GenAI increases well-being, daily creativity, and perceived self-efficacy at work. This link between use and productivity also highlights the value of constant practice as a mechanism for technological adaptation and learning (Agrawal et al., 2022; Raisch and Krakowski, 2021).

In line with this, the positive perception that graduates have about the usefulness of AI in improving their job opportunities reinforces the optimism raised by authors such as Chui et al. (2023) and Kemp (2024), who affirm that AI can increase human capital and generate competitive advantages through intelligent automation. However, this vision is not homogeneous. Studies such as those by Monod et al. (2024) and Golgeci et al. (2025) have warned that many organizations are implementing AI top-down, without adequately considering ethical aspects or the development of human capabilities, which generates distrust, resistance, or ineffective use of these technologies. Some graduates in this study maintain neutral or distrustful positions toward AI, which may be related to fears about surveillance, algorithmic bias, or loss of autonomy, aspects also pointed out by Bankins et al. (2024a), Bankins et al. (2024b), and Wirtz et al. (2023) as key barriers to the ethical adoption of AI in work environments.

Likewise, the generational gap shown in the study, where more recent graduates have higher levels of knowledge, confirms the observations of Fletcher and Nielsen (2024) and Stein et al. (2024), who identify a clear correlation between age, technological familiarity, and AI adoption. This generational difference becomes a structural risk if it is not accompanied by continuous training and professional updating strategies, especially for graduates from previous cohorts. As proposed by Lyndgaard et al. (2024) and Chowdhury et al. (2024), lifelong learning must be part of the institutional design to avoid labor segmentation based on digital obsolescence.

The finding that 95.6% of graduates wish to continue learning about AI is extremely valuable. It not only reinforces what Pavlik (2023) and Atluri et al. (2024) have suggested regarding workers’ willingness to adapt to technological changes, but also highlights a significant opportunity for universities and training centers to implement targeted and accessible training programs. This training demand confirms the need to strengthen the lifelong learning ecosystem (Seeber et al., 2020), where institutions not only certify knowledge but also support the constant updating required by the evolution of AI.

The principal component analysis (PCA) revealed how artificial intelligence (AI) competencies relate to the employability of university graduates, highlighting two key dimensions, one focused on the practical and strategic use of AI, and another on technical knowledge and perceived preparedness. The first dimension, encompassing variables such as the perceived importance of AI, its application in the workplace, and the expectation of improved job opportunities, aligns with the arguments of authors such as Bankins et al. (2024a), Bankins et al. (2024b), Chui et al. (2023), and Jia et al. (2024), who agree that effective AI adoption enhances productivity, boosts creativity, and translates into a tangible competitive advantage. This is also consistent with Kong et al. (2023), who found that a positive relationship with emerging technologies strengthens long-term career sustainability. In contrast, the second dimension, linked to self-reported AI knowledge and perceived readiness to face digital labor market changes, exposes a persistent training gap, as noted by Dwivedi et al. (2021), Reina et al. (2025), and Liu et al. (2024), who point out that many universities have yet to integrate AI comprehensively into their curricula, leaving students dependent on self-directed learning through digital platforms (Fletcher and Nielsen, 2024).

This disconnect between technical proficiency and its effective workplace application can generate feelings of insufficiency, even among highly skilled individuals, echoing Lent et al. (2022) and Hirschi (2018), who emphasize the role of self-efficacy and proactivity in sustaining careers in rapidly evolving contexts. Thus, the PCA shows that the impact of AI on employability is not solely determined by technical knowledge, but also by attitudinal factors, prior experiences, and the professional environment in which it is applied, supporting the claims of Raisch and Krakowski (2021) and Agrawal et al. (2022) that the real value of AI arises when it is strategically embedded in work practices, enabling meaningful collaboration between human and digital capacities. Ultimately, these findings underscore the urgent need for universities not only to teach how to use AI but also to prepare graduates to interpret, contextualize, and lead with it critically, ethically, and with a change-oriented mindset, as advocated by Shneiderman (2022) and Selenko et al. (2022).

While this study emphasizes the positive correlation between AI skills and employability, it is also essential to consider the ethical and practical risks associated with the widespread integration of AI in the workplace. One of the most frequently cited concerns is job displacement due to automation, particularly in routine and low-skilled roles, which may disproportionately affect vulnerable populations (Eloundou et al., 2023; Frey and Osborne, 2017). Furthermore, algorithmic decision-making in recruitment, performance evaluations, or task assignment can reinforce biases embedded in historical data, resulting in unfair outcomes and a lack of transparency (Belanche et al., 2024; Wirtz et al., 2023).

Another key issue is the expansion of digital surveillance, where AI tools are used to monitor employees’ activities, productivity, or even emotions, potentially undermining autonomy and trust (Monod et al., 2024). In parallel, the digital divide remains a persistent structural barrier: individuals without access to training, infrastructure, or connectivity may be left behind in the race toward digital transformation (Golgeci et al., 2025; Liu et al., 2024). These risks highlight the need for educational institutions and organizations not only to promote AI competencies but also to incorporate ethical literacy, inclusive policies, and regulatory frameworks that protect workers’ rights and ensure equitable opportunities. A more balanced perspective on the implications of AI adoption is essential for designing responsible and sustainable strategies for the future of work.

This study provides valuable and context-specific evidence on the association between artificial intelligence (AI) competencies and employability within the setting of a public Peruvian university. However, as with any descriptive and exploratory research, certain methodological limitations must be considered when interpreting the results. The use of a non-probabilistic convenience sample from a single institution may restrict the external validity of the findings, as participants likely share similar institutional, technological, and socioeconomic characteristics (Bornstein et al., 2013; Etikan, 2016). These conditions can influence both access to AI tools and perceptions of professional preparedness. Moreover, potential confounding variables, such as prior work experience, socioeconomic status, or field of study, were not controlled for, which may affect the interpretation of the correlational results (Babbie, 2021; Shadish et al., 2003). Although the study identified associations consistent with findings from other regions, such as perceived increases in productivity and enhanced employability linked to AI use (Jia et al., 2024; Voigt and Strauss, 2024) its cross-sectional design does not allow for causal inferences. These methodological considerations do not diminish the relevance of the findings but rather underscore the need for future research to include broader and more diverse samples, adopt longitudinal or experimental designs, and conduct inter-institutional or cross-cultural comparisons to strengthen both the internal and external validity of studies on AI and employability (Donald et al., 2024).

In summary, the empirical evidence presented here converges with a growing body of literature that underscores that artificial intelligence is not only transforming “what” we do but also “how,” “who,” and “why” we work (Raisch and Krakowski, 2021; Shneiderman, 2022; Sowa et al., 2021). This transformation should not be understood solely as a threat but as an urgent call to rethink the role of higher education, curriculum design, and public employability policies in the era of automation and collaborative intelligence.

## 5 Conclusion

The findings of this study offer a clearer view of how artificial intelligence skills are beginning to play a significant role in the perceived employability of university graduates. While most respondents perceive AI as a valuable and transformative tool, not all have received formal training enabling them to fully leverage its potential. As a result, many graduates report relying on self-study and online platforms, demonstrating initiative in response to the limited structured preparation provided by educational institutions.

The results suggest that those who report higher levels of AI knowledge and more frequent use of AI tools are more likely to report greater job opportunities, better alignment with their careers, and a more positive perception of their productivity. Despite this, a gap persists between generations, with more recent graduates showing greater mastery of these technologies. This underscores the urgent need for universities to integrate AI as a transversal competency across all degree programs, rather than as specialized content for a few disciplines.

The majority of graduates believe that AI will increase their career opportunities and are willing to continue their training in this field, which represents a significant opportunity for universities and continuing education centers. However, it is also evident that there are still doubts, resistance, and a widespread feeling of being “somewhat prepared,” which reinforces the importance of supporting the technological transformation process with solid, ethical training strategies adapted to the new working realities.

From a social and educational policy perspective, the results also highlight the need for profound reform of current educational models. The fact that more than 87% of graduates believe AI should be a structural component of all university programs suggests that this is no longer a technological demand, but rather a social requirement with implications for equity, access to employment, and educational justice. If universities do not update their curricula, they risk widening the digital and employment gaps between those who can access AI training and those who are excluded from the system. Therefore, change must not only be curricular but also supported by public policies that promote educational innovation, investment in technology, and teacher training.

Artificial intelligence skills are not only an added value to a graduate’s professional profile but are also becoming a key requirement for remaining competitive in a rapidly changing labor market. AI is no longer a future scenario; it is present, and being prepared to coexist and work with it is a shared responsibility among graduates, universities, policymakers, and society as a whole. Only through a comprehensive, ethical, and inclusive approach will it be possible to build an education truly aligned with the challenges of the 21st century.

Another important limitation of this study lies in its exclusive reliance on self-reported data, which may introduce biases such as social desirability or overestimation of AI-related competencies. Perceptions of preparedness, productivity, and skill level may not accurately reflect actual performance or objective measures of employability. To address this issue, future research should incorporate more rigorous and objective methods of assessment, such as standardized skills tests, performance-based evaluations, or triangulation with supervisor feedback or work outcomes. These approaches would help validate self-perceptions, reduce bias, and provide a more robust and accurate understanding of the relationship between AI competencies and employability.

One of the limitations of this study is that its descriptive and cross-sectional design prevents the establishment of causal relationships between AI competencies and employability, limiting the depth of the analysis. In this regard, we suggest that future research adopt mixed and longitudinal approaches that allow for observing the evolution of AI’s impact over time. It would also be pertinent to expand the analysis to other universities and socioeconomic contexts, as well as explore in greater depth the ethical, emotional, and cognitive implications arising from human-AI interaction. Finally, we recommend that universities review and update their curricula, incorporating AI as a transversal competency, supported by teacher training programs and institutional strategies that ensure inclusive and effective implementation.

## Data Availability

The original contributions presented in the study are included in the article/Supplementary material, further inquiries can be directed to the corresponding author.
