# Supplementary material for: Artificial intelligence skills and their impact on the employability of university graduates
Source: Front Artif Intell. 2025 Jul 16;8:1629320. doi: 10.3389/frai.2025.1629320 (PMC12307348; doi:10.3389/frai.2025.1629320)
Supplement: Supplementary file 1 [file Supplementary_file_1.pdf]

"Artificial Intelligence Skills and Their Impact on the Employability of University Graduates"

Survey:

Instructions: Please answer the following questions truthfully. The information will be used for research purposes only.

Section 1: General Information
Age:
Gender: ☐Male ☐Female
University Degree: ________________________
Year of graduation: _______________

1. Current employment status:
   - Working in the sector related to my career
   - Working in a sector unrelated to my career
   - Looking for a job
   - Entrepreneurship
   - Other: __________

2. Since you graduated, which of the following situations best describes your work experience?
   - I have been employed most of the time.
   - I have alternated periods of employment and unemployment.
   - I have only worked for short periods of time.
   - I have never worked until now.
   - Own business

3. What do you consider to have been the most decisive factor in getting a job?
   - University education
   - Previous experience or internship
   - Specializations
   - Network of contacts or recommendations
   - I have never worked until now.
   - Other: __________

Section 2: Level of Knowledge in Artificial Intelligence

4. Have you received training in Artificial Intelligence?
   - Yes, in my university career
   - Yes, through external courses or certifications
   - Yes, self-taught.
   - No, I have not received training in AI.

5. What level of knowledge do you think you have in Artificial Intelligence?
   - None
   - Basic (I know the fundamental concepts)
   - Intermediate (I can apply AI tools in my field)
   - Advanced (development or implementation of AI solutions)

6. Which of the following AI tools do you know or have used? (You can select more than one.) *(*Check all that apply)
   - ChatGPT (OpenAI)
   - Microsoft Copilot (integrated with Office 365)
   - Google Gemini (formerly Bard)
   - Perplexity
   - Meta AI
   - Cici AI
   - Gamma
   - Grammarly AI
   - PDF AI
   - I have not used AI tools

7. To what extent do you consider AI knowledge to be important for your professional field?
   - Nothing important
   - Unimportant
   - Important
   - Very important

Section 3: Application of AI in the Workplace

8. How often do you use Artificial Intelligence tools in your work?
   - Very frequently (Every day)
   - Frequently (Several times a week)
   - Occasionally (A few times a month)
   - Seldom
   - I never use them

9. Have you received training in AI?
   - Yes, formally (course, workshop, certification)
   - Yes, self-taught
   - No, but I would like to receive training.
   - No, and I don't consider it necessary to train in AI.

10. Do you think the use of AI has improved your work productivity?
    - Yes, significantly
    - Yes, to some extent
    - It has had no impact on my productivity
    - I don't use AI in my work.

11. How confident are you in using AI tools in your workplace?
    - Very high, I feel comfortable using them
    - High, but I'm still learning
    - Medium, I use them occasionally
    - Low, I have a hard time understanding or applying them
    - I don't use AI tools in my work.

Section 4: Perception of the Relationship between AI and Employability

12. Do you think AI knowledge will increase your job opportunities in the future?
    - Yes, to a large extent
    - Probably
    - I'm not sure
    - I don't believe it

13. How prepared do you feel to face changes in the labor market due to Artificial Intelligence?
    - Very prepared
    - Something prepared
    - Poorly prepared
    - Nothing prepared

14. Do you think universities should strengthen AI teaching across all majors?
    - Yes, it is essential
    - It depends on the race
    - I don't think it's necessary

15. Would you be willing to take a specialization course in AI to improve your employability?
    - Yeah
    - No
    - I'm not sure
